# The therapeutic effect of nicotinamide riboside chloride on ameliorating alcohol-induced neuronal damage with a focus on mitochondrial unfolded protein response and mitophagy

**DOI:** 10.1016/j.gendis.2025.101886

**Published:** 2025-10-18

**Authors:** Jinyang Wang, Jianan Wang, Wei Xie, Qi Shen, Chengbin Wang, Ruibing Li, Shixiong Deng

**Affiliations:** a Department of Forensic Medicine, Chongqing Medical University, Chongqing 400016, China; b Department of Laboratory Medicine, The First Medical Centre of Chinese PLA General Hospital, Beijing 100853, China; c Department of Neurology, The First Medical Centre of Chinese PLA General Hospital, Beijing 100853, China

**Keywords:** Alcohol, Mitophagy, Neuron, Nicotinamide riboside chloride, Unfolded protein response

## Abstract

Excessive alcohol consumption leads to neurodegeneration, driven primarily by oxidative stress and mitochondrial dysfunction, yet no specific treatment exists. Nicotinamide riboside chloride (NRC), a nicotinamide adenine dinucleotide precursor, has demonstrated therapeutic potential in mitigating mitochondrial dysfunction in heart failure, but its role in alcohol-induced neurodegeneration remains unexplored. This study investigated NRC's neuroprotective effects using behavioral tests, serum ethanol and inflammatory marker analysis, hematoxylin-eosin staining, and molecular assays of *in vitro* models. Proteomics and GEO database analysis further elucidated the mechanisms of alcohol-induced brain injury. Results showed that NRC significantly improved alcohol-related cognitive impairment and neuroinflammation. Both our experimental data and external datasets identified mitochondrial dysfunction as a key driver of alcohol-induced neuronal damage, characterized by impaired mitophagy and disrupted mitochondrial unfolded protein response (UPR^mt^). NRC supplementation restored mitochondrial homeostasis by enhancing UPR^mt^ and Fundc1-dependent mitophagy. Mechanistically, UPR^mt^ inhibition abolished NRC's protective effects by suppressing Fundc1 expression and mitophagy, whereas mitophagy inhibition did not affect UPR^mt^, suggesting a hierarchical regulation where UPR^mt^ governs Fundc1-mediated mitophagy. In conclusion, alcohol disrupts mitochondrial quality control, but NRC counteracts neuronal toxicity by activating UPR^mt^ and restoring Fundc1-driven mitophagy, offering a promising therapeutic strategy for alcohol-related neuronal damage.

## Introduction

Alcohol use is recognized as a leading risk factor for the global burden of disease, especially among people aged 15–49.^1^ A Chinese epidemiological study has shown that high alcohol intake is associated with an increased risk of alcoholic dementia.^2^ A history of alcohol consumption or alcohol use disorders is implicated in approximately 5.2% of early-onset dementia cases.^3^ Heavy alcohol consumption may lead to structural and functional damage in the hippocampus, accompanied by neurocognitive dysfunction and memory deficits. These effects are strongly associated with an increased risk of alcoholic dementia.^4^^,^^5^ The pathophysiological processes in the hippocampus induced by alcohol exposure may include oxidative stress, neuroinflammation, and mitochondrial dysfunction.^4^^,^^5^

Mitochondria, as the primary sites of energy generation in the central nervous system, provide stable energy supplies for neuronal growth, regeneration, and functional activities, as well as for various glial cell functions.^6^^,^^7^ Accumulating evidence has demonstrated that normal axonal ion channel activities and synaptic signal transmission depend on mitochondrial energy supply.^8^, ^9^, ^10^ By impairing axonal and synaptic function, mitochondrial dysfunction contributes to the pathogenesis of neurodegenerative diseases. However, alcohol primarily affects the regulation of ion channels and receptors involved in synaptic transmission and neuronal excitability.^11^ This interference disrupts synapse formation and neurotransmission. These findings suggest that mitochondrial dysfunction may represent a underlying pathogenic mechanism in alcohol-related cognitive disorders.^12^, ^13^, ^14^

Mitophagy and the mitochondrial unfolded protein response (UPR^mt^) are two essential quality control processes that actively maintain mitochondrial homeostasis by eliminating misfolded proteins and damaged organelles.^15^^,^^16^ When mitochondria are subjected to oxidative stress and aging, the loss of membrane potential activates mitophagy. This process promptly removes senescent and damaged mitochondria to maintain normal cellular function.^17^ Unlike mitophagy, UPR^mt^ is primarily regulated by proteostasis.^18^ This pathway maintains mitochondrial protein quality control by monitoring the status of molecular chaperones and transcription factors.^18^ The accumulation of misfolded proteins, a hallmark pathological feature of neurodegenerative diseases, impairs mitochondrial protein quality control. This impairment is a major contributor to the progression of these diseases.^19^ However, the involvement of the UPR^mt^ in the pathogenesis of neurodegenerative diseases remains unclear. In Parkinson's disease studies, activation of mitophagy accelerated the clearance of damaged mitochondria, reduced reactive oxygen species (ROS) accumulation, decreased oxidative stress, and thus exerted neuroprotective effects.^20^ These findings, taken together, suggest a probable association between alcohol-induced neuronal damage and abnormalities in UPR^mt^ and mitophagy. However, no conclusion on the causal interaction between UPR^mt^ and mitophagy has been drawn, and the underlying molecular mechanisms remain unclear.

The fact that nicotinamide riboside chloride (NRC) has an inductive and activating effect on UPR^mt^,^21^ combined with the absence of reported side effects from its supplementation, makes it particularly valuable for research. Furthermore, neurons are more susceptible to alcohol-induced apoptosis compared with other glial cells, exhibiting notable changes in mitochondrial structure and quality control imbalances.^22^ In cases of chronic brain injury, NRC supplementation can provide energy to the brain, reduce hippocampal infarct volume, and contribute to neuronal protection.^23^ Additionally, NRC supplementation exerts neuroprotective effects by maintaining mitochondrial structural integrity.^24^ Therefore, NRC therapy shows promise as a neuroprotective agent and may have therapeutic potential in brain injury. Based on this, the present study aimed to further elucidate the neurotoxic effects and pathological mechanisms of alcohol on the central nervous system, while exploring the therapeutic value of NRC supplementation in protecting neurons from alcohol-induced damage.

## Materials and methods

### Animal model and evaluation

All materials were presented in the Supplementary materials. Male C57BL/6 mice (9–13 weeks) were obtained from SPF Biotechnology Co., Ltd. (Beijing, China). The mice were randomly assigned to four groups: negative control (NC) group, alcohol group, NRC group, and alcohol plus NRC group (12 mice per group, 3 per cage), with cage positions systematically controlled to avoid potential bias. The sample size was calculated based on the survival rate of the primary endpoint and the pilot data. Due to differences in feeding and drug administration methods between groups, the experimenters could not be blinded to the treatment assignments. However, at the end of the study, histopathological analyses and behavioral tests were independently assessed by two additional researchers who were blinded to group assignments. All mice were housed in the temperature-controlled environment under a 12 h/12 h light/dark cycle, and were randomly assigned to four individual cages (NC group, NRC group, alcohol group, alcohol plus NRC group; *n* = 12 in each group). The alcohol-treated group received continuous alcohol administration for 4 weeks.^25^ NRC (350 mg/kg·d, MCE, USA) was supplemented continuously for two weeks until the end of the experiment, as previously described.^26^^,^^27^ Blood samples were collected to measure alcohol concentration and inflammatory markers (IL-6 and MCP-1), while brain tissues were analyzed for mitochondrial ATP levels, mitochondrial complex IV activity, and ultrastructural morphology. Moreover, hematoxylin-eosin staining was used to visualize alcohol-induced brain damage, and the shortest vertical distance between the upper and lower edges of the dentate gyrus was measured using ImageJ (measurement points are indicated by arrows in the figure). Mice were excluded based on the following criteria: low blood alcohol concentration, no significant abnormalities in behavioral assessments, absence of notable pathological damage, and mortality or poor health status during the experiment.

### Behavioral assessment

To observe the effects of alcohol exposure and NRC treatment on the cognitive abilities of mice, the open field test and Morris water maze test were used to detect behavioral changes in mice. Total distance traveled in the open field, time spent in the central zone, and average velocity were recorded according to the protocol of the previous study.^28^^,^^29^ For the water maze experiment, mice were required to be trained continuously for 5 days before their memory was examined. The detailed protocol for training and probe trials was listed in the Supplementary materials.

### Transmission electron microscopy

As described previously,^30^ mouse hippocampus was fixed in 4% polyformaldehyde for 24 h. Next, the tissues were washed, dehydrated, and embedded, then polymerized in a 60 °C oven for 48 h. The wax blocks were subsequently sectioned (60–80 nm thickness) using an ultramicrotome, stained with uranyl acetate and lead citrate, and examined under a transmission electron microscope to observe mitochondrial morphology and measure their length. Mitochondrial length was quantified using ImageJ.

### Cellular proteomic analysis

Cells from both alcohol-treated and control groups (*n* = 3 biological replicates per condition) were harvested and lysed using RIPA buffer supplemented with complete protease inhibitor cocktail. The extracted proteins were denatured in an 8 M urea solution. The extracted proteins underwent sequential processes including disulfide bond reduction, alkylation, trypsin digestion, desalting (C18 column), and lyophilization. The peptides were reconstituted in 0.1% formic acid and quantified using a Nanodrop spectrophotometer (Thermo Fisher Scientific, USA). Peptide separation was performed on an Ultimate 3000 nanoLC system (Thermo Fisher Scientific) coupled to a Q-Exactive HF mass spectrometer (Thermo Fisher Scientific) operating in positive ion mode. Data were acquired using data-dependent acquisition.

The raw mass spectrometry data were processed using Proteome Discoverer 2.4 against the Mus musculus UniProt database. Protein abundance data were normalized and statistically analyzed using Perseus software (version 1.6.15.0). The normalized proteomic data were analyzed to identify significantly differentially expressed proteins (*P* < 0.05, |fold-change| > 1.5). Multi-dimensional bioinformatics approaches comprising heatmap visualization of expression patterns, Gene Ontology (GO) functional annotation, and Gene Set Enrichment Analysis (GSEA) systematically revealed the predominant biological processes underlying alcohol-mediated neural damage. Enrichment analysis was conducted to interpret the relevant biological functions (Wei Sheng Xin, https://www.bioinformatics.com.cn/; OECloud, https://cloud.oebiotech.cn). GO analysis was conducted with the ClusterProfiler R package. The analysis relied on the package's default hypergeometric test and multiple testing correction.

### External dataset acquisition and analysis

The Gene Expression Omnibus (GEO, http://www.ncbi.nlm.nih.gov/geo) database was queried to explore the molecular mechanisms of alcohol-induced brain injury. The GSE154934 database was used as an external dataset for further study. Differentially expressed genes were selected with a condition of *P* < 0.05 and |fold-change| > 1.2. We performed the comprehensive functional enrichment analyses to identify the potential functions of differentially expressed genes using GO. GSEA was performed using transcriptomics data from the dataset, and GSEA data results were filtered according to *P* < 0.05 (OECloud, https://cloud.oebiotech.cn; detailed analysis results are presented in the Supplementary materials). Further analysis of differentially expressed genes intersecting genes with mitochondria and autophagy datasets was facilitated by the STRING database (https://cn.string-db.org/) and visualized using Cytoscape 3.10.3.

### Cell culture and treatments

The HT22 cells were cultured in HT22-specific culture medium at 37 °C with 5% CO_2_. All *in vitro* experiments were conducted at 50%–60% cell density (unless stated otherwise), a condition optimized through preliminary studies to balance cell viability, proliferation rate, and prevention of contact inhibition. Through measuring cell viability, we determined the effective concentrations of alcohol (150 mM), NRC (750 μM), AEBSF (100 μM), and MF094 (5 μM). HT22 cells were incubated with alcohol for 48 h. NRC, AEBSF, and MF094 were separately added to the HT22-specific culture medium 6 h before alcohol model construction. HT22 cells were transfected with siRNAs (GenePharma, Shanghai) by Lipofectamine RNAiMAX transfection reagent (Thermo Fisher, USA).

### Cell apoptosis assay

After alcohol treatment for 48 h, the apoptosis was performed with the use of a one-step TUNEL apoptosis assay kit (Beyotime, China). The experimental steps were carried out according to the instructions. Images were captured, and the relative fluorescence intensity of apoptotic cells was semi-quantified using ImageJ.

### CCK-8 assay

HT22 cells were plated in 96-well plates and maintained at 37 °C for 24 h. Experimental groups (including alcohol-treated samples) and control groups were established, with five replicate wells per group. Following the administration of various drug treatments as required, cell viability was assessed. After model establishment, 10 μL of CCK-8 solution (Tohjin Co., Japan) was added to each well, followed by a 2-h incubation at 37 °C in the dark. Absorbance at 450 nm (optical density) was then measured using a microplate reader (Thermo Fisher, USA). Cell survival rates were calculated using the following formula: survival rate = (optical density of experimental group/optical density of control group) × 100%.

### Western blotting

Western blotting was performed as described previously.^31^ Primary antibodies included FUN14 domain-containing 1 (Fundc1; CST, #49240), light chain 3 (LC3; CST, #12741), Tubulin (Huaxingbio, HX1829), mitochondrial complex IV (COX IV; Huaxingbio, #HX1842), interleukin 6 (IL6; CST, #12912), Caspase9 (CST, #9508), B-cell lymphoma 2 (Bcl2; CST, #3498), and Bcl2-associated X (Bax; CST, #14796). Secondary antibodies (goat anti-rabbit/mouse IgG conjugated with horseradish peroxidase, 1:10,000, HX2061, HX2062) were purchased from Huaxingbio, Beijing, China. The relative optical density values of each protein were semi-quantified using ImageJ software.

### Real-time quantitative PCR

According to the manufacturer's instructions, real-time quantitative PCR was used to observe the expression of UPR^mt^-related genes after extracting the RNA and synthesizing the cDNA through reverse transcription. GAPDH served as the internal control. Gene expression levels in hepatocytes from different groups were quantified using the 2^–ΔΔCt^ method. Details of the primers used in our study are provided in Table 1.

**Table 1:** Primer sequences (mouse).

Primer name	Upstream primer (5′–3′)	Downstream primer (5′–3′)
*GAPDH*	ACGGCAAATTCAACGCACAGTCA	TGGGGGCATCGGCAGAAGG
*mtDNAj*	AGTCACCCACACAAGCACTG	CCAGCCTCTCGCCTATCC
*Atf5*	TCCGCTCACACCGTCTCT	AAGGCGAAGGTGGAGGAC
*Lonp1*	GGTTGAGAATGTAGCCCATGA	CGATGATATCCCGAATGGTC
*Fundc1*	GGCUAUGUACAGAUCGACUTT	AGUCGAUCUGUACAUAGCCTT

### Mitochondrial functional assays

Mitochondrial function was evaluated by measuring mitochondrial permeability transition pore, mitochondrial membrane potential, mitochondrial ROS, mitochondrial kinetics, autophagosome number, ATP content, and mitochondrial complex IV activity. For each experimental condition, ≥ 3 random fields were imaged from 3 independent biological replicates (total *n* = 9 images/group). All images were captured using an Olympus FV1000 laser confocal microscope; meanwhile, mitochondrial length and fluorescence intensity values were semi-quantitatively analyzed using ImageJ (v1.53, NIH). Details of the experiments are shown in the supplementary materials.

### Statistical analysis

Data were presented as mean ± standard deviation. SPSS 19.0 and GraphPad Prism 8 were used for statistical analysis. All experiments were independently repeated at least three times, each consisting of three biological replicates that were normalized and averaged to generate a single data point per experiment. The Shapiro–Wilk test and Brown–Forsythe test were performed to assess data normality and variance homogeneity, respectively. Normal distribution data were compared between groups using one-way analysis of variance (ANOVA). When data did not meet these parametric assumptions, non-parametric tests (*e.g.*, Mann–Whitney *U* or Kruskal–Wallis test) were employed. A *P*-value less than 0.05 was considered statistically significant.

## Results

### NRC mitigated alcohol-induced cognitive impairments

**Figure 1:**
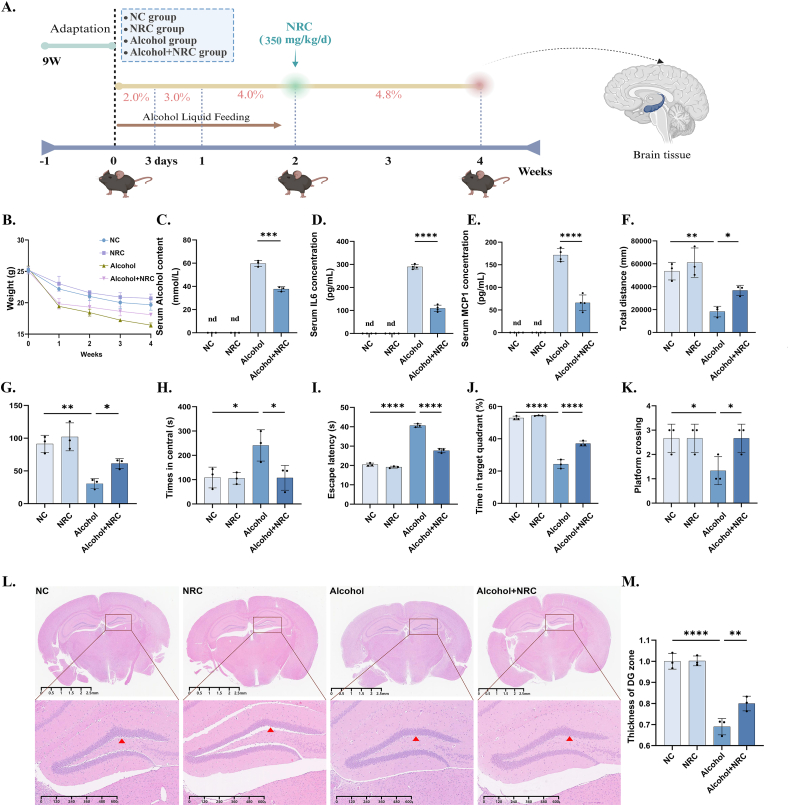
Nicotinamide riboside chloride (NRC) supplement blunted the development of neuron damage in alcohol-induced mice. **(A)** Experimental protocol. All animals were divided into 4 groups: negative control (NC) group, NRC group, alcohol group, and alcohol plus NRC group. Alcohol gradient feeding was conducted for 4 weeks. 2 weeks after alcohol gradient feeding, mice were treated with NRC (350 mg/kg·d) for 2 weeks. **(B)** The weight changes of mice under 4 weeks of control/alcohol liquid feeding. **(C)** Serum alcohol content. **(D, E)** Serum IL6 and MCP1 levels. **(F–K)** Behavior test. The mice were subjected to an open field test and Morris water maze test, described as total distance (F), average velocity (G), times in the central (H), escape latency (I), platform crossing (J), and time in the target quadrant (K). **(L)** Four weeks later, the mice were sacrificed, and the hippocampus was collected. Hematoxylin-eosin staining was used to observe the alcohol-derived brain damage. Data quantification was shown in Figure 1M. Each group included three mice, with three independent replicates performed. All data were presented as mean ± standard deviation. *P*-values were determined using a two-tailed unpaired *t*-test. ns, not significant; nd, non-detected; ∗*P* < 0.05, ∗∗*P* < 0.01, ∗∗∗*P* < 0.001, and ∗∗∗∗*P* < 0.0001.

To monitor the survival status of mice under alcohol exposure and NRC treatment, body weight was measured weekly for over 4 weeks. The results indicated that mice subjected to 4 weeks of alcohol consumption exhibited significant weight loss compared with the control group (Fig. 1A and B). In response to alcohol exposure, blood analysis revealed elevated alcohol content and a three-fold increase in inflammatory factors (Fig. 1C–E). Nonetheless, continuous NRC supplementation effectively prevented alcohol-induced weight loss and suppressed the systemic inflammatory response (Fig. 1B–E).

Subsequently, behavioral assessments revealed that alcohol exposure impaired cognitive function in mice. Alcohol-fed mice exhibited a decrease in the total moved distance, slower movement speed, and longer resting time in the central area (Fig. 1F–H), as well as reduced time spent in the target quadrant and fewer crossings over the original platform after its removal (Fig. 1I–K). In contrast, NRC improved exploration and learning abilities (Fig. 1F–K), suggesting that NRC administration ameliorated alcohol-induced brain damage and cognitive deficits. Hematoxylin-eosin staining also confirmed NRC's protective effects on hippocampal structure, demonstrating that treatment reversed alcohol-induced thinning of the dentate gyrus (Fig. 1L and M).

### NRC attenuated alcohol-induced cellular inflammation and apoptosis

**Figure 2:**
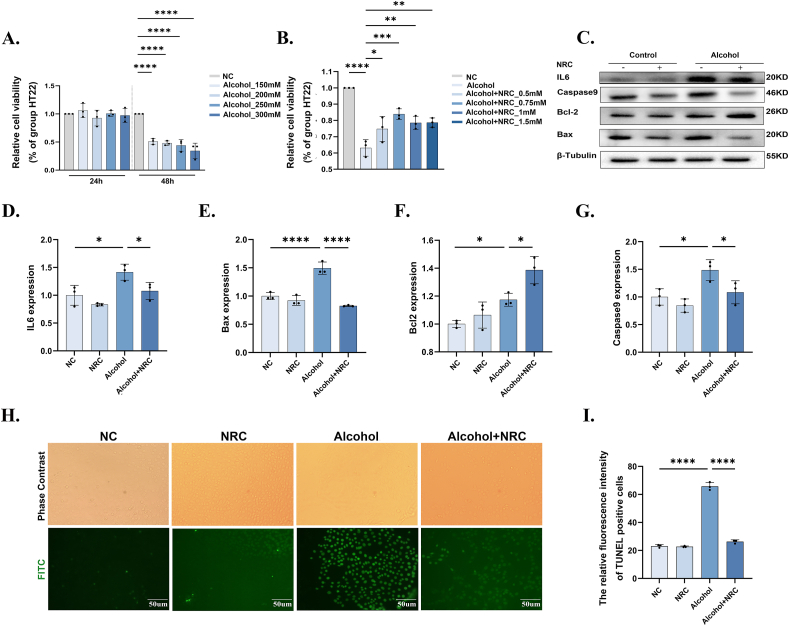
Nicotinamide riboside chloride (NRC) attenuates alcohol-induced neuron injury. **(A)** The effect of different culture times (24 h, 48 h) and different alcohol concentrations (0 mM, 150 mM, 200 mM, 250 mM, 300 mM) on neuronal cell vitality. **(B)** Under the condition of 150 mM alcohol stimulation for 48 h, we selected the optimal concentrations of NRC to reduce the alcohol-induced damage. **(C)** Western blotting was used to detect the expression of inflammatory and apoptosis proteins among four groups: negative control (NC) group, NRC group, alcohol group, and alcohol plus NRC group. **(D**–**G)** Quantitative analysis of inflammatory and injury-related protein expression in the four groups. **(H, I)** Levels of apoptosis were assessed by TUNEL staining and were quantified by ImageJ. Each value was expressed as mean ± standard deviation (*n* = 3 independent experiments). ∗*P* < 0.05, ∗∗*P* < 0.01, ∗∗∗*P* < 0.001, and ∗∗∗∗*P* < 0.0001.

To further elucidate the molecular mechanisms underlying alcohol's effects, we performed CCK8 assays and selected 150 mM alcohol for 48 h to establish an *in vitro* model of alcohol-induced neuronal damage (Fig. 2A). In parallel, we selected 750 μM NRC using CCK8 assays for subsequent experiments (Fig. 2B). Then, we found that alcohol exposure exacerbated the inflammation and apoptosis processes, as evidenced by elevated expression of IL6, Caspase9, and Bax proteins, as well as decreased expression of Bcl2 protein (Fig. 2C–G). Subsequently, levels of apoptosis were assessed via TUNEL staining, which revealed a significant increase in integrated fluorescence intensity in the alcohol group, indicating elevated cell death (Fig. 2H and I). Notably, NRC supplementation could reduce inflammation protein expression and alleviate cell damage in the setting of alcohol.

Integrated *in vitro* and *in vivo* studies demonstrated that NRC supplementation effectively ameliorated alcohol-induced inflammation response and provided protective effects.

### Gene expression profiling revealed dysregulation of mitochondrial function and UPR^mt^ in alcohol-induced neurotoxicity

**Figure 3:**
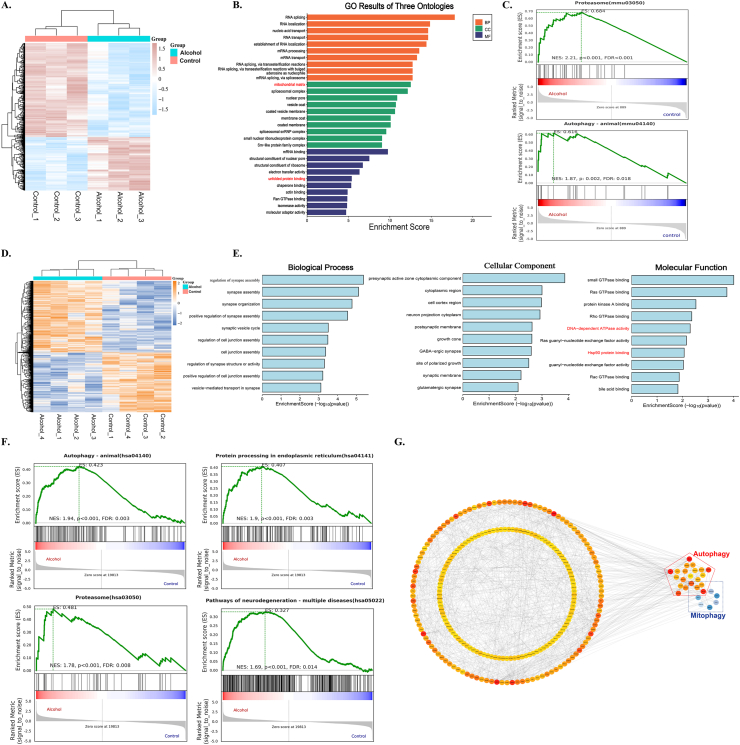
Enrichment analysis for the main mechanisms of action of alcohol-induced neurotoxicity. Using mass spectrometry, we investigated alcohol-induced proteomic alterations in cells, including heatmap analysis **(A)**, Gene Ontology (GO) enrichment analysis **(B)**, and Gene Set Enrichment Analysis (GSEA) **(C)**. Subsequently, these findings were validated using the GSE154934 dataset. Alcohol-induced neurotoxicity was mimicked using human induced pluripotent stem cell-derived three-dimensional brain-like organoids, observed after 6 h of alcohol/control exposure, and brain RNA was extracted for heatmap and GO analysis **(D, E)**. **(F)** GSEA showed that alcohol may cause a disorder between mitophagy and UPR^mt^, and an increased risk of cognitive impairment. **(G)** Protein–protein interaction network. The differential protein interaction network was based on the STRING database and visualized by Cytoscape.

To further investigate the molecular mechanisms of alcohol-induced neurotoxicity, we used mass spectrometry to analyze proteomic changes in cells exposed to alcohol. As shown in Figure 3A, a total of 377 differentially expressed proteins were identified (Control *vs.* Alcohol, *P* < 0.05, fold change > 1.5). GO analysis revealed that these differentially expressed proteins primarily affected the mitochondrial matrix and unfolded protein binding (Fig. 3B). GSEA showed that alcohol-induced neural injury mainly occurred through influencing biological processes related to the proteasome and autophagy (Fig. 3C).

Subsequently, to further validate the above findings, we normalized arrays from the GSE154934 dataset and identified 1505 differentially expressed genes in cerebral organoids treated with and without alcohol (*P* < 0.05, |fold change| > 1.2) (Fig. 3D). GO analysis revealed that differentially expressed genes were primarily involved in synapse assembly, presynaptic active zone cytoplasmic component, DNA-dependent ATPase activity, and HSP90 protein binding (Fig. 3E). GSEA indicated that alcohol-induced neurotoxicity was significantly enriched in pathways related to autophagy, protein processing in the endoplasmic reticulum, proteasome function, and neurodegeneration (Fig. 3F). The protein–protein interaction network showed that 20 genes were associated with autophagy, of which 4 genes were involved in the mitophagy pathway (Fig. 3G).

Based on these findings, we hypothesized that alcohol-induced neurotoxicity primarily resulted from impaired mitochondrial function, particularly mitophagy, and activation of UPR^mt^.

### NRC ameliorated mitochondrial oxidative stress and maintained mitochondrial integrity in the setting of alcohol injury

**Figure 4:**
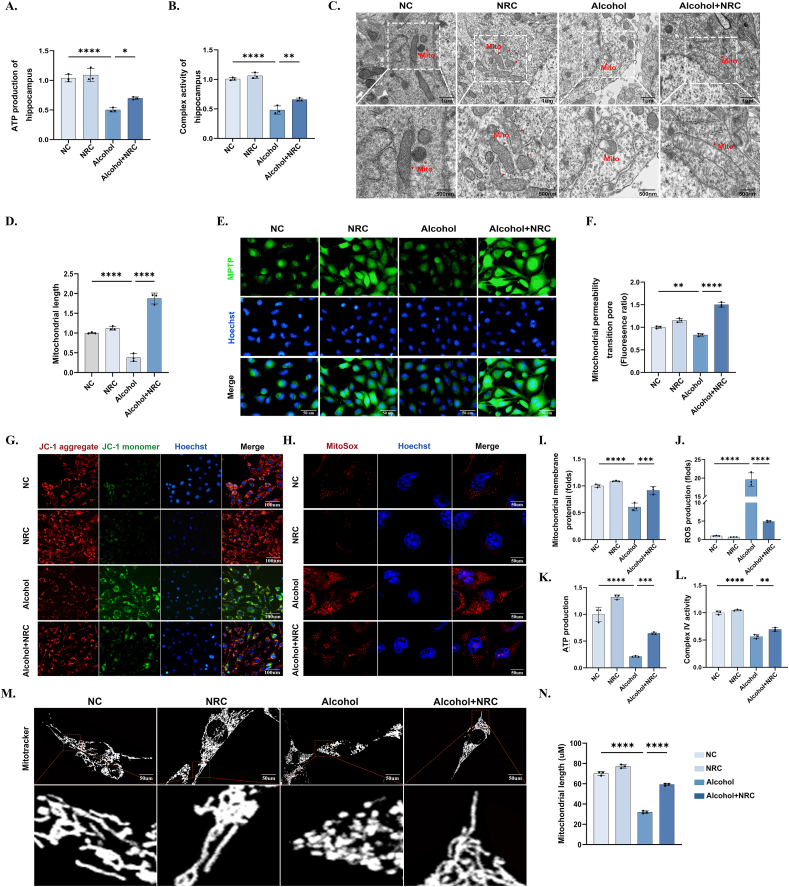
Nicotinamide riboside chloride (NRC) treatment ameliorated mitochondrial dysfunction and mitochondrial oxidative stress. **(A)** ATP content of the mouse hippocampus. **(B)** Mitochondrial complex IV activity of the mouse hippocampus. **(C, D)** Transmission electron microscopy analysis of mitochondrial alterations in mouse brain tissue following alcohol exposure and NRC supplementation. **(E, F)** Significant improvement in mitochondrial permeability transition pore (MPTP) was observed after NRC treatment. **(G, I)** Mitochondrial membrane potential. **(H, J)** Mitochondrial ROS production. **(K)** ATP content of HT22 cells. **(L)** Mitochondrial complex IV activity of HT22 cells. **(M, N)** Mitochondrial integrity was used to detect changes in mitochondrial dynamics in the negative control (NC) group, NRC group, alcohol group, and the alcohol plus NRC group, and the average length of mitochondria in cells was measured (scale bar: 50 μm). The results were shown by the representative images and their corresponding quantitative results. Each group included three mice, with three independent replicates performed. Each value was expressed as mean ± standard deviation (*n* = 3 independent experiments). ∗*P* < 0.05, ∗∗*P* < 0.01, ∗∗∗*P* < 0.001, and ∗∗∗∗*P* < 0.0001.

Mitochondrial function assessments in the cerebrum showed reduced ATP content and complex IV activity in alcohol-fed mice, which were partially restored by NRC supplementation (Fig. 4A and B). In addition, transmission electron microscopy revealed significant alterations in mitochondrial morphology in the brain tissue of alcohol-exposed mice, including marked swelling, loss of cristae, reduced quantity, and shortened length (Fig. 4C and D). Notably, NRC intervention substantially ameliorated these abnormalities, restoring the mitochondria to their typical elongated rod-shaped morphology, increasing their number, and promoting length extension (Fig. 4C and D). These findings suggest that NRC supplementation may exert a restorative effect on alcohol-induced mitochondrial damage in brain tissue. Molecular assays showed that alcohol exposure increased mitochondrial permeability transition pore opening and disrupted mitochondrial membrane potential (Fig. 4E–G, I). Furthermore, alcohol induced oxidative stress in neuronal mitochondria, leading to elevated accumulation of ROS, reduced ATP synthesis, and impaired complex IV activity (Fig. 4H, J–L). NRC supplementation effectively ameliorated these mitochondrial dysfunctions, as evidenced by decreased membrane depolarization, reduced ROS accumulation, restored ATP production, and improved mitochondrial complex IV activity.

Since mitochondria are essential for energy supply for neuronal synaptic transmission, mitochondrial dysfunction could be a primary reason for alcohol-induced neuronal damage.^32^ Our findings suggested that alcohol disrupted mitochondrial homeostasis, leading to mitochondrial fragmentation and impaired function. As shown in Figure 4K, mitochondria exhibited an elongated rod-shaped distribution in the NC group. However, alcohol exposure induced mitochondrial fragmentation, as evidenced by an increased number of smaller, fragmented mitochondria (Fig. 4M and N). NRC treatment reversed these effects, promoting mitochondrial elongation and restoring a rod-shaped morphology.

### NRC restored neuronal mitochondrial integrity by promoting UPR^mt^ and mitophagy

**Figure 5:**
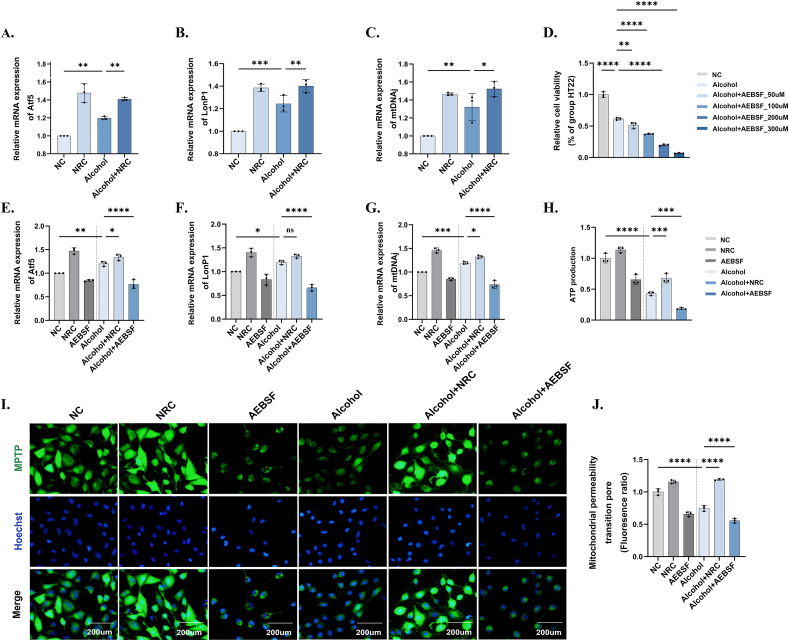
Nicotinamide riboside chloride (NRC) supplement reversed mitochondrial integrity via UPR^mt^. **(A**–**C)** Real-time quantitative PCR assay was applied to analyze UPR^mt^ gene expression (*Atf5*, *mtDNAj*, *LonP1*) under the role of NRC. **(D)** 100 μM AEBSF (UPR^mt^ inhibitor) was considered the optimal concentration to inhibit cell viability. **(E**–**G)** Real-time quantitative PCR assay was applied to analyze UPR^mt^ gene expression (*Atf5*, *mtDNAj*, *LonP1*) under the role of AEBSF and NRC. **(H)** Effect of AEBSF on ATP content. **(I, J)** Significant improvement in mitochondrial permeability transition pore (MPTP) was observed after NRC and AEBSF treatment. Quantitative analysis of mitophagy proteins and mitochondrial dynamic proteins was performed, with each value expressed as mean ± standard deviation (*n* = 3 independent experiments). NC, negative control. ns, no significant; ∗*P* < 0.05, ∗∗*P* < 0.01, ∗∗∗*P* < 0.001, and ∗∗∗∗*P* < 0.0001.

Given NRC's role as an inducer of UPR^mt^,^21^ we investigated the expression of UPR^mt^ markers using real-time quantitative PCR. Compared with the alcohol-only group, NRC supplementation further activated UPR^mt^ (Fig. 5A–C). To determine whether UPR^mt^ activation conferred neuroprotective effects, the UPR^mt^ antagonist AEBSF was introduced into the cell culture before alcohol exposure.^33^ As shown in Figure 5D, 100 μM AEBSF was confirmed due to cellular reduction by half under the alcohol exposure. Meanwhile, AEBSF significantly inhibited the expression of *Atf5*, *mtDNAj*, and *LonP1* (Fig. 5E–G), while these markers of UPR^mt^ were up-regulated in the alcohol plus NRC group. Further observation showed that AEBSF suppressed the production of mitochondrial ATP and increased the levels of mitochondrial permeability transition pore opening, further augmenting the mitochondrial dysfunction in neurons under alcohol stimulation (Fig. 5H–J).

**Figure 6:**
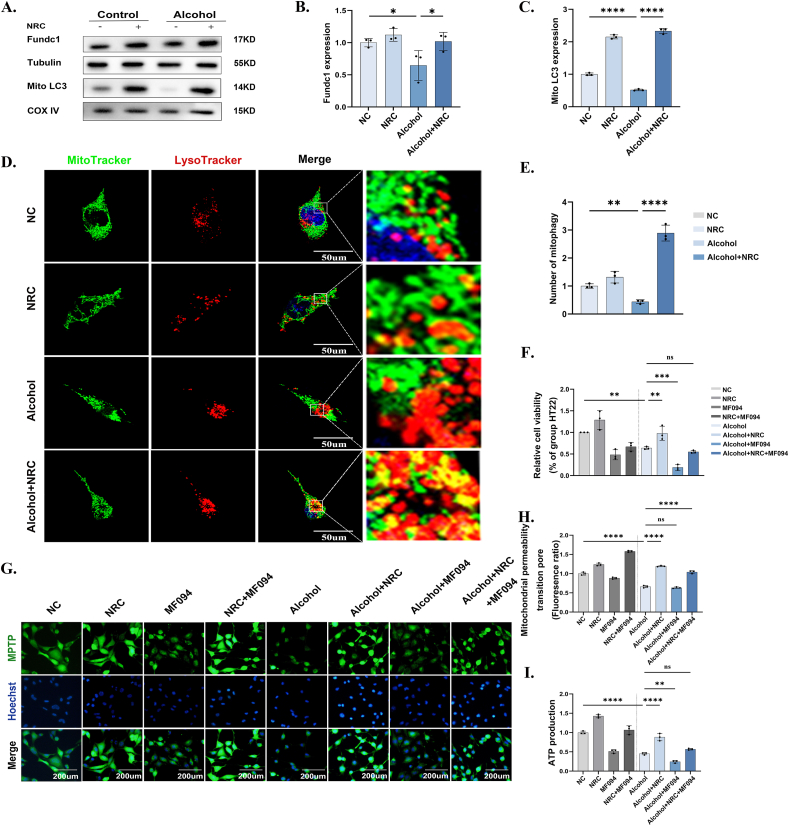
Nicotinamide riboside chloride (NRC) restored mitochondrial function through mitophagy. **(A**–**C)** Western blotting of mitophagy protein (Fundc1, Mito-LC3) expression treated with NRC. **(D, E)** The co-immunofluorescence of mitochondria and lysosomes indicated mitophagy. **(F)** Cell viability assays of HT22 cells treated with 5 μM MF094 (mitophagy inhibitor) for 6 h before alcohol exposure. **(G**–**I)** Effect of mitophagy inhibition on ATP content and mitochondrial permeability transition pore. Each value was expressed as mean ± standard deviation (*n* = 3 independent experiments). ns, no significant; ∗*P* < 0.05, ∗∗*P* < 0.01, ∗∗∗*P* < 0.001, and ∗∗∗∗*P* < 0.0001.

Furthermore, our study indicated that NRC increased the expression of mitophagy proteins Fundc1 and Mito-LC3 (Fig. 6A–C). NRC treatment under alcohol exposure further promoted mitochondrial and lysosomal interactions, robustly activating mitophagy, the selective removal of damaged mitochondria or mitochondrial fragments (Fig. 6D and E). NRC not only regulated UPR^mt^ to restore neuronal mitochondrial function but also enhanced mitophagy activity. The information suggests that mitophagy may be required for NRC's neuroprotection. Since a previous study has shown that MF094 can inhibit mitophagy by promoting the ubiquitination of Fundc1.^34^ Therefore, in the present study, HT22 cells were treated with MF094 (5 μM) 6 h before alcohol exposure to inhibit mitophagy (Fig. 6F). Data showed that MF094 abolished the protective effects of NRC, impairing ATP production, and increasing mitochondrial membrane permeability (Fig. 6H and I).

Altogether, our data indicate that UPR^mt^ and mitophagy are drastically affected by alcohol and are reactivated by NRC to protect neurons against stress.

### Inhibition of UPR^mt^ interfered with mitochondrial function by affecting mitophagy

Based on these results, it was evident that alcohol induced neurotoxicity mainly by interfering with mitophagy and UPR^mt^, whereas NRC supplementation significantly activated mitophagy and enhanced UPR^mt^, thereby exerting a protective effect on neurons exposed to alcohol.

**Figure 7:**
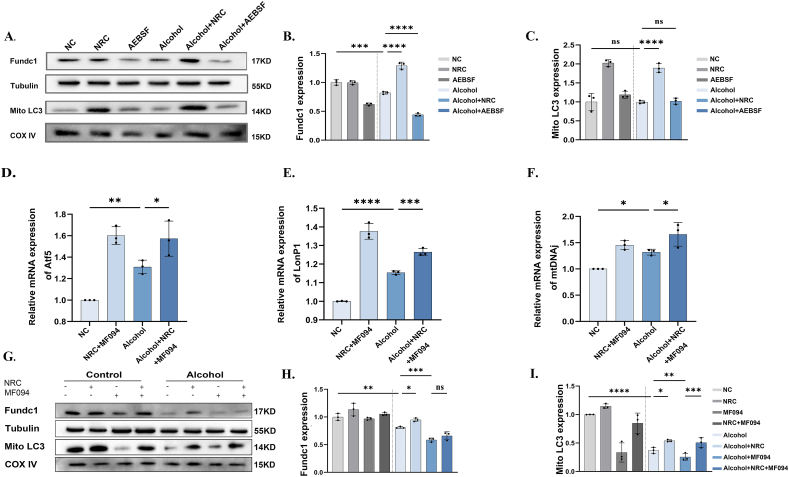
Nicotinamide riboside chloride (NRC) supplement reduced the alcohol-induced neuronal injury by affecting Fundc1-mediated mitophagy. **(A**–**C)** Western blotting of the mitophagy protein (Fundc1, Mito-LC3) expression treated with AEBSF and NRC. **(D**–**F)** Real-time quantitative PCR. NRC was able to elevate the mRNA levels of *Atf5*, *mtDNAj*, and *LonP1* upon mitophagy inhibition. **(G**–**I)** Western blotting of the mitophagy protein (Fundc1, Mito-LC3) expression treated with MF094 and NRC. Each value was expressed as mean ± standard deviation (*n* = 3 independent experiments). NC, negative control. ns, no significant; ∗*P* < 0.05, ∗∗*P* < 0.01, ∗∗∗*P* < 0.001, and ∗∗∗∗*P* < 0.0001.

To investigate the relationship between UPR^mt^ and mitophagy, we observed their change in response to inhibitors. Notably, AEBSF inhibited the expression of mitophagy proteins (Fig. 7A–C). In addition, further examination of mRNA expression of UPR^mt^-related genes showed that NRC was still able to elevate the mRNA levels of *Atf5*, *LonP1*, and *mtDNAj* when inhibiting mitophagy (Fig. 7D–F). Interestingly, we found that NRC failed to reverse Fundc1-dependent mitophagy in response to MF094 (Fig. 7G–I), suggesting that UPR^mt^ may exert neuroprotection primarily by affecting Fundc1-dependent mitophagy.

**Figure 8:**
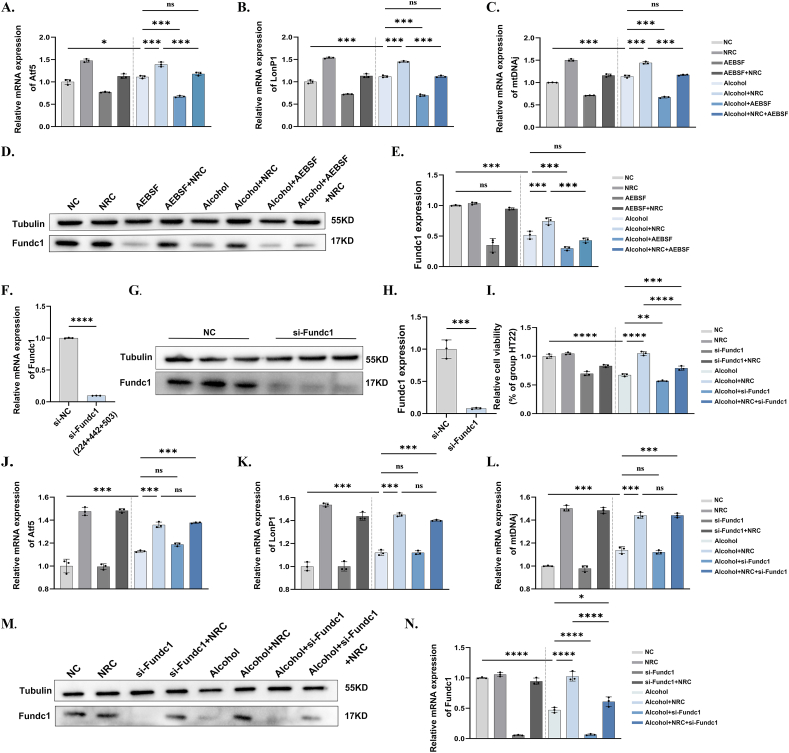
Fundc1-mediated mitophagy is a critical pathway in alcohol-induced neurotoxicity and functions downstream of UPR^mt^. We concurrently treated cells with both UPR^mt^ activators and inhibitors to examine **(A**–**C)** expression changes of *Atf5*, *mtDNAj*, and *LonP1* genes, and **(D, E)** alterations in Fundc1 protein levels. **(F–H)** Validation of *Fundc1* knockdown efficiency. **(I)** CCK8 assay for effects of *Fundc1* co-knockdown on HT22 cell viability. **(J**–**L)** Real-time quantitative PCR analysis of UPR^mt^-related gene (*Atf5*, *mtDNAj*, *LonP1*) expression following Fundc1 knockdown. **(M, N)** Western blot analysis of Fundc1 protein expression with NRC supplementation after *Fundc1* knockdown. Each value was expressed as mean ± standard deviation (*n* = 3 independent experiments). NC, negative control. ns, no significant; ∗*P* < 0.05, ∗∗*P* < 0.01, ∗∗∗*P* < 0.001, and ∗∗∗∗*P* < 0.0001.

Subsequently, we simultaneously activated and inhibited UPR^mt^ or knocked down *Fundc1* to investigate whether the Fundc1-mediated mitophagy pathway served as the primary critical pathway in alcohol-induced neurotoxicity. The results showed that co-treatment with NRC (UPR^mt^ activator) and AEBSF (UPR^mt^ inhibitor) counteracted the effects of *Atf5/mtDNAj/LonP1* activation or inhibition (Fig. 8A–C). Moreover, compared with alcohol exposure alone, the expression of Fundc1 protein in the alcohol/NRC/AEBSF group showed no significant changes (*t* = 1.953, degrees of freedom = 16, *P* = 0.35; Fig. 8D and E). To further validate these findings, we knocked down Fundc1 expression using pooled siRNAs and confirmed the knockdown efficiency (Fig. 8F–H). The CCK8 assay revealed that Fundc1 knockdown significantly reduced the viability of HT22 cells under alcohol exposure, an effect that could be rescued by NRC supplementation (Fig. 8I). Results also demonstrated that under alcohol exposure conditions, *Fundc1* knockdown showed no significant effect on UPR^mt^-related gene expression (alcohol group *vs*. alcohol plus si-Fundc1 group; Atf5: *t* = 2.499, degrees of freedom = 16, *P* = 0.11; mtDNAj: *t* = 0.22, degrees of freedom = 16, *P* > 0.99; LonP1: *t* = 0.98; degrees of freedom = 16, *P* = 0.88) (Fig. 8J–L). However, additional NRC supplementation in the alcohol plus Fundc1 knockdown group significantly up-regulated the expression levels of UPR^mt^-related genes (Fig. 8J–L), while also markedly increasing Fundc1 protein expression (Fig. 8M and N).

Therefore, our results suggested that UPR^mt^ was located upstream of Fundc1-induced mitophagy, and the axis played a vital protective role against alcohol-induced neuronal damage.

## Discussion

The brain, as one of the primary target organs for alcohol, is adversely affected by alcohol exposure.^35^^,^^36^ This exposure particularly damages neurons in critical regions, such as the hippocampus and cortex, an effect that is associated with the development of neurodegenerative diseases.^37^^,^^38^ Our results showed that alcohol impaired the cognitive abilities of mice, which was ameliorated with NRC treatment. Hematoxylin-eosin staining data revealed that alcohol primarily reduced hippocampus thickness and that NRC treatment may attenuate alcohol-induced hippocampal injury. Our data and the GEO database suggest that mitochondrial dysfunction may be a primary mechanism of alcohol-induced neuronal damage. NRC supplementation can activate UPR^mt^ to alleviate neuronal toxicity caused by alcohol and reduce neuronal damage by regulating Fundc1-mediated mitophagy.

Mitochondria distribution varies across different types of cells.^39^^,^^40^ In neurons, mitochondria are predominantly concentrated in the soma and dendritic regions, forming an extensive dynamic network that provides an immediate supply of ATP to support neuronal activities, thereby enhancing their functional performance.^41^ In contrast, mitochondria are less abundant in the axonal regions of neurons, which increases their susceptibility to oxidative damage.^42^^,^^43^ Consequently, even minor metabolic impairments or disruptions in energy homeostasis abnormalities can significantly impact neuronal function.^6^^,^^42^ Given that the central nervous system is a primary target of alcohol damage, we hypothesize that alcohol may impair neuronal function by disrupting mitochondrial activity. Our study found that alcohol exposure triggered severe mitochondrial dysfunctions, including decreased membrane potential, impaired mitophagy, ROS accumulation, and abnormal mitochondrial dynamics. Additionally, another study indicates that mitochondrial oxidative stress in the zebrafish brain can be induced under both acute and chronic ethanol exposure, leading to increased ROS production.^44^ *In vivo* experiments have also shown cognitive decline in rats exposed to alcohol, as indicated by shorter moving distances in the open field test.^45^ These findings confirm that alcohol induces mitochondrial dysfunction in neurons, which contributes to neurodegenerative changes. Therefore, to prevent neurodegenerative development caused by alcohol abuse, we aim to further explore neuroprotective strategies and the underlying molecular mechanisms involved.

External databases revealed that alcohol primarily affects mitophagy and UPR^mt^ in the brain, potentially increasing the risk of neurodegenerative diseases. To validate this finding, we employed an *in vitro* model to demonstrate impairment of mitochondrial function in response to alcohol exposure. Results showed that alcohol stimulation exacerbated oxidative stress in neurons, resulting in significant ROS accumulation, mitochondrial permeability transition pore opening, loss of membrane potential, mitochondrial fragmentation, and marked inhibition of mitophagy. Mitophagy, as one of the crucial molecular pathways for maintaining cellular and organismal homeostasis, can eliminate damaged mitochondria and bear a “protective” role in the body.^46^ Clearly, alcohol suppressed the expression of mitophagy proteins, indicating that the alcohol-induced neuronal damage exceeded the recovery capacity of mitophagy. However, impaired mitophagy negatively affects cellular health and induces or exacerbates disease progression.^47^ Dysfunctional mitophagy and lysosomal pathways fail to clear cellular debris effectively, leading to the excessive deposition of amyloid-beta peptides and tau proteins, which ultimately trigger Alzheimer's disease.^48^^,^^49^ Therefore, promoting mitophagy may be an effective strategy for preventing neurodegenerative diseases or age-related diseases. In this study, NRC supplementation promoted mitochondrial and lysosomal interactions, significantly increased the expression of mitophagy proteins, and reduced alcohol damage to neurons. A similar study also demonstrated that Fundc1-required mitophagy maintained mitochondrial structure and function, and that its absence exacerbated mitochondrial damage.^46^ This finding underscored the crucial role of Fundc1-related mitophagy in safeguarding mitochondrial integrity. UPR^mt^ activation is recognized as an adaptive protection and dynamically maintains intracellular homeostasis, especially in response to mitochondrial dysfunction.^50^ Our data revealed that although alcohol exposure induced a modest up-regulation of UPR^mt^, this endogenous response was insufficient to maintain mitochondrial integrity or support cell viability. We hypothesize that alcohol elicits an adaptive activation of UPR^mt^ as a compensatory mechanism against external stress. However, this activation remains inadequate to confer substantial cytoprotection. In contrast, supplementation with NRC further potentiated UPR^mt^-related gene expression and concurrently promoted mitophagy, ultimately leading to robust neuroprotection. Notably, under conditions of UPR^mt^ activation, NRC reversed alcohol-induced neuronal mitochondrial damage by further promoting Fundc1-associated mitophagy. This provided new insights and therapeutic targets for addressing alcohol-induced neuronal damage. Currently, there is a certain limitation in our study. Although current evidence supports the position of UPR^mt^ upstream of Fundc1-mediated mitophagy, future studies utilizing a mitophagy activator (such as metformin or Pink1/Parkin modulators) under UPR^mt^-inhibited conditions would help to further corroborate the proposed hierarchy within this pathway. Furthermore, proteomic analysis was conducted on alcohol- and NRC-treated HT-22 cells to investigate potential mechanistic pathways under controlled *in vitro* conditions. However, to strengthen the translational relevance of these findings, future studies should include *in vivo* validation of the key pathways identified herein. Moreover, it is important to note that the dosage and therapeutic efficacy of NRC may vary due to individual and ethnic differences, which challenges the generalizability of our results.

## Conclusion

Our findings suggest that NRC regulates mitochondrial abnormalities through a UPR^mt^-mitophagy interaction mechanism, which helps to treat alcohol-induced brain injury and provides neuronal protection. This study also offers insights for the clinical translation and application of mitochondrial-targeted drugs in neurons.

## CRediT authorship contribution statement

**Jinyang Wang:** Writing – original draft, Validation, Methodology. **Jianan Wang:** Validation, Formal analysis, Data curation. **Wei Xie:** Validation, Data curation. **Qi Shen:** Resources, Investigation. **Chengbin Wang:** Writing – review & editing, Project administration, Conceptualization. **Ruibing Li:** Writing – review & editing, Supervision, Methodology. **Shixiong Deng:** Writing – review & editing, Project administration, Conceptualization.

## Data availability

The mass spectrometry proteomics data have been deposited to the ProteomeXchange Consortium (https://proteomecentral.proteomexchange.org) with the dataset identifier PXD064283.

## Ethics declaration

Animal experimental procedures were approved by the Institutional Animal Care and Use Committee of Chinese PLA General Hospital (Approval ID: 2020-x16-78; Date: 5 August 2020). Animal care was performed in accordance with the Basel Declaration, “Guide for the Care and Use of Laboratory Animals” 8th Edition and the guidelines of the International Council for Laboratory Animal Science (ICLAS).

## Funding

This research was primarily supported by Chongqing Graduate Student Research Innovation Project (No. CYB23192, Chongqing, China) and Independent Innovation Scientific Project of Chinese PLA General Hospital (No. 22QNCZ058, Beijing, China).

## Conflict of interests

The authors declared no competing interests.

## References

[1] Di Castelnuovo A.F., Costanzo S., de Gaetano G. (2019). Alcohol and the global burden of disease. Lancet.

[2] Millwood I.Y., Im P.K., Bennett D. (2023). Alcohol intake and cause-specific mortality: conventional and genetic evidence in a prospective cohort study of 512000 adults in China. Lancet Public Health.

[3] Schwarzinger M., Pollock B.G., Hasan O.S.M., Dufouil C., Rehm J. (2018). Contribution of alcohol use disorders to the burden of dementia in France 2008-13: a nationwide retrospective cohort study. Lancet Public Health.

[4] Niedzwiedz-Massey V.M., Douglas J.C., Rafferty T., Wight P.A., Kane C.J.M., Drew P.D. (2021). Ethanol modulation of hippocampal neuroinflammation, myelination, and neurodevelopment in a postnatal mouse model of fetal alcohol spectrum disorders. Neurotoxicol Teratol.

[5] Vore A.S., Deak T. (2022). Alcohol, inflammation, and blood-brain barrier function in health and disease across development. Int Rev Neurobiol.

[6] Pekkurnaz G., Wang X. (2022). Mitochondrial heterogeneity and homeostasis through the lens of a neuron. Nat Metab.

[7] Cheng X.T., Huang N., Sheng Z.H. (2022). Programming axonal mitochondrial maintenance and bioenergetics in neurodegeneration and regeneration. Neuron.

[8] Jordán J., Ceña V., Prehn J.H. (2003). Mitochondrial control of neuron death and its role in neurodegenerative disorders. J Physiol Biochem.

[9] Harris J.J., Jolivet R., Attwell D. (2012). Synaptic energy use and supply. Neuron.

[10] Cardanho-Ramos C., Morais V.A. (2021). Mitochondrial biogenesis in neurons: how and where. Int J Mol Sci.

[11] Abrahao K.P., Salinas A.G., Lovinger D.M. (2017). Alcohol and the brain: neuronal molecular targets, synapses, and circuits. Neuron.

[12] Mira R.G., Tapia-Rojas C., Pérez M.J. (2019). Alcohol impairs hippocampal function: from NMDA receptor synaptic transmission to mitochondrial function. Drug Alcohol Depend.

[13] Casañas-Sánchez V., Pérez J.A., Quinto-Alemany D., Díaz M. (2016). Sub-toxic ethanol exposure modulates gene expression and enzyme activity of antioxidant systems to provide neuroprotection in hippocampal HT22 cells. Front Physiol.

[14] Tapia-Rojas C., Mira R.G., Torres A.K. (2017). Alcohol consumption during adolescence: a link between mitochondrial damage and ethanol brain intoxication. Birth Defects Res.

[15] Zhu L., Luo X., Fu N., Chen L. (2021). Mitochondrial unfolded protein response: a novel pathway in metabolism and immunity. Pharmacol Res.

[16] Gottlieb R.A., Piplani H., Sin J. (2021). At the heart of mitochondrial quality control: many roads to the top. Cell Mol Life Sci.

[17] Zhao Y., Zhou L., Li H. (2021). Nuclear-encoded lncRNA MALAT1 epigenetically controls metabolic reprogramming in HCC cells through the mitophagy pathway. Mol Ther Nucleic Acids.

[18] Weng H., Ma Y., Chen L. (2020). A new vision of mitochondrial unfolded protein response to the sirtuin family. Curr Neuropharmacol.

[19] Xu S., Liu H., Wang C. (2023). Dual roles of UPR(er) and UPR(mt) in neurodegenerative diseases. J Mol Med (Berl).

[20] Prasertsuksri P., Kraokaew P., Pranweerapaiboon K., Sobhon P., Chaithirayanon K. (2023). Neuroprotection of andrographolide against neurotoxin MPP^+^-induced apoptosis in SH-SY5Y cells via activating mitophagy, autophagy, and antioxidant activities. Int J Mol Sci.

[21] Gariani K., Menzies K.J., Ryu D. (2016). Eliciting the mitochondrial unfolded protein response by nicotinamide adenine dinucleotide repletion reverses fatty liver disease in mice. Hepatology.

[22] Arzua T., Yan Y., Jiang C. (2020). Modeling alcohol-induced neurotoxicity using human induced pluripotent stem cell-derived three-dimensional cerebral organoids. Transl Psychiatry.

[23] Cheng Y.H., Zhao J.H., Zong W.F. (2022). Acute treatment with nicotinamide riboside chloride reduces hippocampal damage and preserves the cognitive function of mice with ischemic injury. Neurochem Res.

[24] Wang L., Peng T., Deng J. (2024). Nicotinamide riboside alleviates brain dysfunction induced by chronic cerebral hypoperfusion via protecting mitochondria. Biochem Pharmacol.

[25] Zhou H.Z., Karliner J.S., Gray M.O. (2002). Moderate alcohol consumption induces sustained cardiac protection by activating PKC-Epsilon and Akt. Am J Physiol Heart Circ Physiol.

[26] Williams A.S., Koves T.R., Pettway Y.D. (2022). Nicotinamide riboside supplementation confers marginal metabolic benefits in obese mice without remodeling the muscle acetyl-proteome. iScience.

[27] Hou Y., Wei Y., Lautrup S. (2021). NAD^+^ supplementation reduces neuroinflammation and cell senescence in a transgenic mouse model of Alzheimer’s disease via cGAS-STING. Proc Natl Acad Sci U S A.

[28] Tan L., Cheng Y., Wang H., Tong J., Qin X. (2022). Peripheral transplantation of mesenchymal stem cells at sepsis convalescence improves cognitive function of sepsis surviving mice. Oxid Med Cell Longev.

[29] Banko J.L., Poulin F., Hou L., DeMaria C.T., Sonenberg N., Klann E. (2005). The translation repressor 4E-BP2 is critical for eIF4F complex formation, synaptic plasticity, and memory in the hippocampus. J Neurosci.

[30] Sun C.C., Chen Z.L., Yang D. (2025). Loss of popdc3 impairs mitochondrial function and causes skeletal muscle atrophy and reduced swimming ability in zebrafish. J Cachexia Sarcopenia Muscle.

[31] Walker M.A., Chen H., Yadav A. (2023). Raising NAD^+^ level stimulates short-chain dehydrogenase/reductase proteins to alleviate heart failure independent of mitochondrial protein deacetylation. Circulation.

[32] Liu Q., Wang X., Hu Y. (2023). Acetylated tau exacerbates learning and memory impairment by disturbing with mitochondrial homeostasis. Redox Biol.

[33] Wang B., Chen W., Huang Q., Chen Y., Wang Y. (2024). Targeting cancer mitochondria by inducing an abnormal mitochondrial unfolded protein response leads to tumor suppression. Int J Med Sci.

[34] Chen G., Chen L., Huang Y., Zhu X., Yu Y. (2022). Increased FUN14 domain containing 1 (FUNDC1) ubiquitination level inhibits mitophagy and alleviates the injury in hypoxia-induced trophoblast cells. Bioengineered.

[35] Fang S.Q., Wang Y.T., Wei J.X., Shu Y.H., Xiao L., Lu X.M. (2016). Beneficial effects of chlorogenic acid on alcohol-induced damage in PC12 cells. Biomed Pharmacother.

[36] Hernandez J., Kaun K.R. (2022). Alcohol, neuronal plasticity, and mitochondrial trafficking. Proc Natl Acad Sci U S A.

[37] Peng B., Yang Q., B Joshi R. (2020). Role of alcohol drinking in Alzheimer's disease, Parkinson's disease, and amyotrophic lateral sclerosis. Int J Mol Sci.

[38] Chandrashekar D.V., Steinberg R.A., Han D., Sumbria R.K. (2023). Alcohol as a modifiable risk factor for Alzheimer's disease: evidence from experimental studies. Int J Mol Sci.

[39] Pathak D., Shields L.Y., Mendelsohn B.A. (2015). The role of mitochondrially derived ATP in synaptic vesicle recycling. J Biol Chem.

[40] Kuznetsov A.V., Hermann M., Saks V., Hengster P., Margreiter R. (2009). The cell-type specificity of mitochondrial dynamics. Int J Biochem Cell Biol.

[41] Li S., Xiong G.J., Huang N., Sheng Z.H. (2020). The cross-talk of energy sensing and mitochondrial anchoring sustains synaptic efficacy by maintaining presynaptic metabolism. Nat Metab.

[42] Rangaraju V., Calloway N., Ryan T.A. (2014). Activity-driven local ATP synthesis is required for synaptic function. Cell.

[43] Hill R.L., Kulbe J.R., Singh I.N., Wang J.A., Hall E.D. (2018). Synaptic mitochondria are more susceptible to traumatic brain injury-induced oxidative damage and respiratory dysfunction than non-synaptic mitochondria. Neuroscience.

[44] Müller T.E., Nunes M.E.M., Rodrigues N.R. (2019). Neurochemical mechanisms underlying acute and chronic ethanol-mediated responses in zebrafish: the role of mitochondrial bioenergetics. Neurochem Int.

[45] Motaghinejad M., Motevalian M., Fatima S., Hashemi H., Gholami M. (2017). Curcumin confers neuroprotection against alcohol-induced hippocampal neurodegeneration via CREB-BDNF pathway in rats. Biomed Pharmacother.

[46] Zhou H., Zhu P., Wang J., Zhu H., Ren J., Chen Y. (2018). Pathogenesis of cardiac ischemia reperfusion injury is associated with CK2α-disturbed mitochondrial homeostasis via suppression of FUNDC1-related mitophagy. Cell Death Differ.

[47] Klionsky D.J., Petroni G., Amaravadi R.K. (2021). Autophagy in major human diseases. EMBO J.

[48] Kerr J.S., Adriaanse B.A., Greig N.H. (2017). Mitophagy and Alzheimer's disease: cellular and molecular mechanisms. Trends Neurosci.

[49] Picca A., Faitg J., Auwerx J., Ferrucci L., D'Amico D. (2023). Mitophagy in human health, ageing and disease. Nat Metab.

[50] Pellegrino M.W., Haynes C.M. (2015). Mitophagy and the mitochondrial unfolded protein response in neurodegeneration and bacterial infection. BMC Biol.

