# Supplementary material for: The therapeutic effect of nicotinamide riboside chloride on ameliorating alcohol-induced neuronal damage with a focus on mitochondrial unfolded protein response and mitophagy
Source: Genes Dis. 2025 Oct 18;13(5):101886. doi: 10.1016/j.gendis.2025.101886 (PMC13276746; doi:10.1016/j.gendis.2025.101886)
Supplement: Multimedia component 1 [file mmc1.docx]

**Supplementary Materials**

1. **Materials**

HT22 cells (Wuhan Pricella Biotechnology, China); Anhydrous ethanol (National Pharmaceutical Group Corporation, China); Nicotinamide riboside chloride (NR) and AEBSF (MCE, US); MF094 (mitophagy inhibitor, Sigma, US); HT22-specific culture medium (Wuhan Pricella Biotechnology, China); Trypsin (Promega, US); RNA extraction kit (Nanjing Nuoweizan Biotechnology, China); cDNA synthesis kit (Bio-RAD, US); primers (Sangon Biotech, Shanghai, China); MitoSox Red and MitoTracker Green (Thermo Fisher Scientific, US); Mitochondrial membrane potential detection (JC-1) kit and Mitochondrial permeability transition pore (MPTP) and 100X Hoechst (Beyotime, China); ATP content assays kit (Nanjing Jiancheng Bioengineering Institute, China); mitochondrial complex IV activity (Solarbio, China).

1. **Behavioral assessment**

**Open Filed Test.** We applied the open filed test for observing locomotor activity in mice. In a word, every mouse was placed at the center place in the apparatus (50×50×50 cm) firstly. The movement trajectory of each mouse was tracked in real time using SuperMaze (XR - Xmaze, Shanghai Xinran), and the total distance traveled, time at rest and explored were recorded. Data was collected for a period 600s. To avoid the odor interference between each mouse, the filed box was cleaned with 75% ethanol after each mouse was used, and leaved it to dry before proceeding to the next one. During the experiment, mice should be gently grasped to avoid artificial interference.

**Morris water maze.** The training paradigm consisted of the hidden platform and the probe trial. The hidden platform training time lasted for 60s each time for 5 consecutive learning days, and the exploration experiment was conducted after the fifth training day for 2 consecutive days. The escape platform was marked with visual clues, and was randomly moved between four positions, and the movement trajectory was recorded with the video tracking system (SuperMaze, Shanghai Xinran).

1. **The measure of ROS**

Experimental groups were divided into four: NC group, NR group, Alcohol group, and Alcohol+NR group. HT22 cells were seeded in confocal dishes (3 dishes per group). After different treatments, cells were gently washed twice with PBS, then incubated with 2 uM MitoSOX red dye per dish at 37°C for 15 minutes in the dark. Subsequently, 1× Hoechst live cell stain was added and incubated at 37°C for 5 minutes. After removing the working solution, cells were washed twice with PBS and observed in fresh HT22 culture medium using an Olympus FV1000 laser confocal microscope. Images were captured.

1. **Mitochondrial membrane potential**

Mitochondrial membrane potential was detected using the JC-1 assay kit and 1× Hoechst live cell stain. After gentle washing with PBS twice, cells were incubated with JC-1 dye at 37°C for 15 minutes in the dark, followed by incubation with 1× Hoechst live cell stain at 37°C for 5 minutes. After removing the working solution, cells were gently washed twice with JC-1 buffer and then imaged under a fluorescence microscope. When mitochondrial membrane potential is maintained, JC-1 aggregates in the mitochondrial matrix, emitting red fluorescence, whereas damaged mitochondria exhibit decreased membrane potential and display green fluorescence. Mitochondrial depolarization was measured using the ratio of red to green fluorescence.

Fluorescence images were semi-quantitatively analyzed for fluorescence intensity using ImageJ software.

1. **Mitochondrial permeability transition pore (MPTP)**

Mitochondrial pore activity was assessed by Mitochondrial permeability transition pore assay kit (Beyotime, China). Cells were incubated in the dark for 30min at 37℃ in 100ul Calcein AM staining solution (1X) and Fluorescence quenching solution (1X). Next, cells were replaced with fresh pre-warmed culture medium at 37℃ for 30min in the dark, and were counterstained with 1× Hoechst. The stained cells were subjected the fluorescence microscope for image analysis. The opening degree of mitochondrial MPTP was determined by the intensity of the green fluorescence of Calcein in the mitochondria, and the stronger the green fluorescence, the lower the degree of opening of the cells.

1. **Assessment of mitochondrial morphology in cells**

This experiment comprised four groups: NC group, NR group, Alcohol group, and Alcohol+NR group. HT22 cells were seeded into confocal dishes (three dishes per group). After different treatments, the cells are gently washed twice with PBS, then stained with MitoTracker probe to observe mitochondrial morphology. Subsequently, they were incubated at 37°C in the dark for 20 minutes, followed by the addition of 1× Hoechst live cell dye and further incubation at 37°C in the dark for 5 minutes. After removing the working solution, the cells are gently washed twice with PBS and then transferred to fresh HT22-specific culture medium for observation. The dishes were observed and photographed under an OLYMPUS FV1000 laser confocal microscope (Japan).

1. **Mitophagy assessment**

This experiment comprised four groups: NC group, NR group, Alcohol group, and Alcohol+NR group. HT22 cells were seeded into confocal dishes (three dishes per group). After different treatments, the cells are gently washed twice with PBS, then stained with MitoTracker probe and LysoTracker probe to observe mitochondrial morphology. Subsequently, they were incubated at 37°C in the dark for 20 minutes, followed by the addition of 1× Hoechst live cell dye and further incubation at 37°C in the dark for 5 minutes. After removing the working solution, the cells are gently washed twice with PBS and then transferred to PBS for observation. The dishes were observed and photographed under an OLYMPUS FV1000 laser confocal microscope.

1. **ATP evaluation**

The ATP assay kit (Nanjing Jiancheng Bioengineering Institute, China) was used to detect ATP levels in hippocampus tissue and HT22 cells. Following the supplier’s instructions, each sample takes 1×10^6^ cells, placed in an ice water bath and uniformly disrupted, and then the protein concentration was measured. 30uL of cells suspension was added to of ATP detection buffer (330uL), incubated at 37℃ for 30 minutes in a water bath, and centrifuged at 4000 rpm for 5 minutes. 30uL supernatant was added to the chromogenic in a 96-well plate. After incubating in the dark for 2 minutes, the absorbance was measured at 636nm with a microplate reader (Thermo Fisher, USA).

1. **Mitochondrial Complex IV activity**

The activity of mitochondrial complex IV was detected using the mitochondrial complex IV activity assay kit (Solarbio, China). Cells and hippocampus tissue were uniformly crushed on ice and the supernatant was retained for Complex IV activity assay. The subsequent experiments were carried out in strict accordance with the instructions. Finally, the data were read at 550nm and used to analyze the changes in mitochondrial complex IV enzyme activity.
